# Supplementary material for: Conditional chemoconnectomics (cCCTomics) as a strategy for efficient and conditional targeting of chemical transmission
Source: eLife. 2024 Apr 30;12:RP91927. doi: 10.7554/eLife.91927 (PMC11060718; doi:10.7554/eLife.91927)
Supplement: Figure 3—source data 3. [file elife-91927-fig3-data3.zip › Figure 3 source data3 part 2.zip!/Figure 3-Source data 3-part2/1060f2-1-CRISPResso_Report_gl5umwyq.zip!/CRISPResso_on_1060f2_1/1c.Alignment_barplot.pdf]

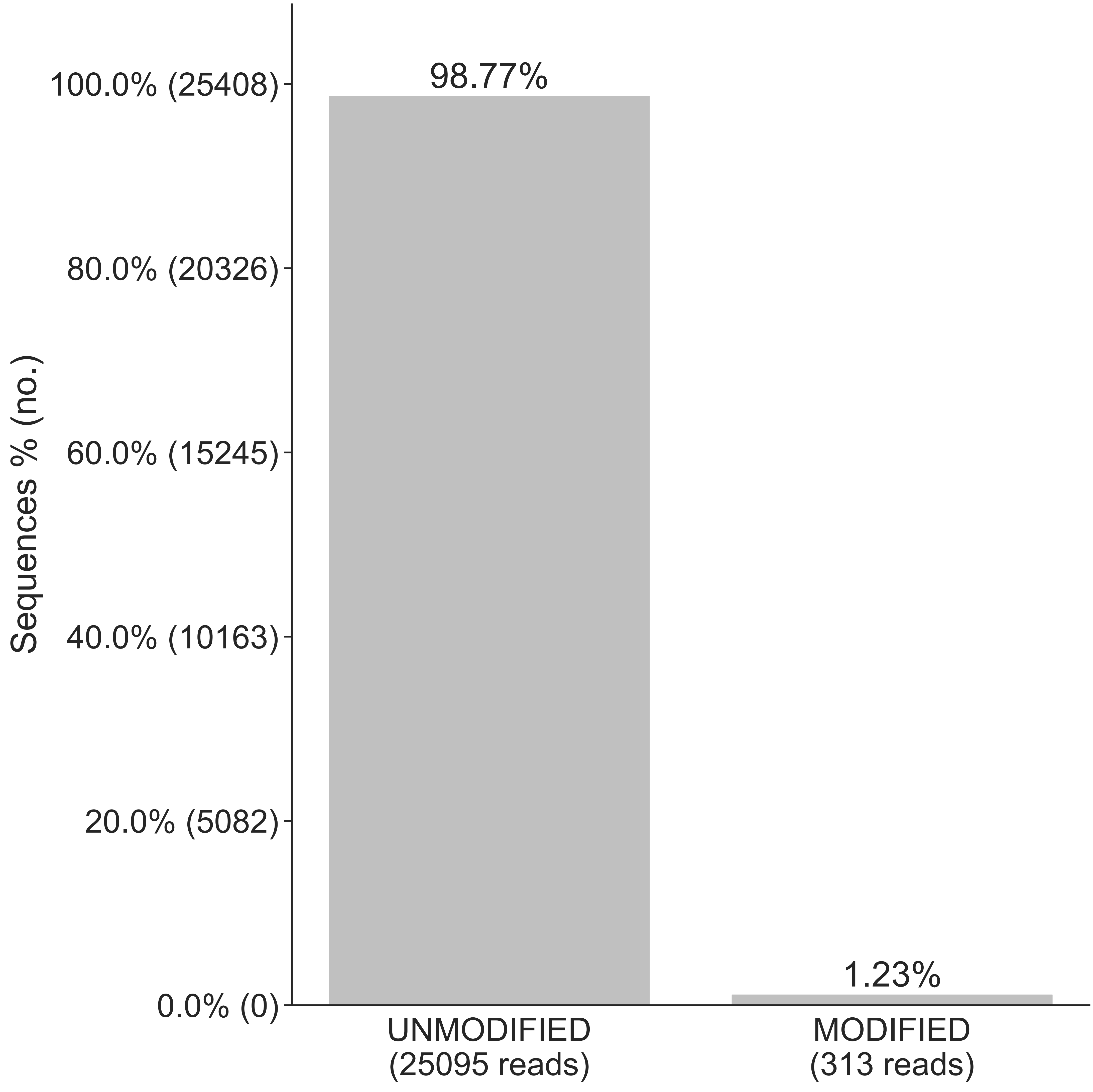
98.77%
1.23%
Sequences % (no.)
100.0% (25408)
80.0% (20326)
60.0% (15245)
40.0% (10163)
20.0% (5082)
0.0% (0)
UNMODIFIED
(25095 reads)
MODIFIED
(313 reads)
